# A Pilot Study of “Peer Navigators” to Promote Uptake of HIV Testing, Care and Treatment Among Street-Connected Children and Youth in Eldoret, Kenya

**DOI:** 10.1007/s10461-018-2276-1

**Published:** 2018-09-29

**Authors:** Pooja Shah, Mia Kibel, David Ayuku, Regina Lobun, John Ayieko, Alfred Keter, Allan Kamanda, Dominic Makori, Collins Khaemba, Anthony Ngeresa, Lonnie Embleton, Katherine MacDonald, Edith Apondi, Paula Braitstein

**Affiliations:** 1 Academic Model Providing Access to Healthcare (AMPATH), Eldoret, Kenya; 2 Department of Epidemiology, Dalla Lana School of Public Health, University of Toronto, 155 College Street, 5th Floor, Toronto, ON M5T 3M7 Canada; 3 Department of Behavioral Sciences, School of Medicine, College of Health Sciences, Moi University, Eldoret, Kenya; 4 Moi Teaching and Referral Hospital, Eldoret, Kenya; 5 Faculty of Medicine, Institute of Medical Science, University of Toronto, Toronto, Canada; 6 Department of Pediatrics, School of Medicine, Indiana University, Indianapolis, USA; 7 Department of Medicine, School of Medicine, College of Health Sciences, Moi University, Eldoret, Kenya

**Keywords:** Peer support, HIV, Youth, Homeless, Africa

## Abstract

Research suggests a burden of HIV among street-connected youth (SCY) in Kenya. We piloted the use of peer navigators (PNs), individuals of mixed HIV serostatus and with direct experience of being street-connected, to link SCY to HIV testing and care. From January 2015 to October 2017, PNs engaged 781 SCY (585 male, 196 female), median age 16 (IQR 13–20). At initial encounter, 52 (6.6%) were known HIV-positive and 647 (88.8%) agreed to HIV testing. Overall, 63/781 (8.1%) SCY engaged in this program were HIV-positive; 4.6% males and 18.4% females (p < 0.001). Of those HIV-positive, 48 (82.8%) initiated ART. As of October 2017, 35 (60.3%) of the HIV-positive SCY were alive and in care. The pilot suggests that PNs were successful in promoting HIV testing, linkage to care and ART initiation. More research is needed to evaluate how to improve ART adherence, viral suppression and retention in care in this population.

## Introduction

Adolescents and youth face particular challenges in accessing services for HIV testing, treatment, and care [1]. Between 2005 and 2012, while the number of AIDS-related deaths fell by 30% globally, HIV-related mortality among adolescents increased by 50% [1]. Youth aged 15–24 currently account for 37% of all new HIV infections in Sub-Saharan Africa (SSA) [2, 3]. HIV testing is the main entry point into treatment and care for people living with HIV, but rates of testing are still inadequate [4]. In Kenya, only half of adolescents aged 15–19 have ever been tested for HIV, though among those aged 20–24, 92% of females and 67% of males have been tested for HIV. Fewer than 50% of adolescents who are HIV-positive are on antiretroviral treatment (ART), and the number retained in care is unclear [5]. ART initiation and retention in care remain prominent challenges in this population [6, 7].

Current targets from The Joint United Nations Programme on HIV/AIDS (UNAIDS) state that by the year 2020, 90% of people living with HIV should know their status, 90% of them should be receiving ART, and 90% of those on ART should have an undetectable viral load [3]. In Kenya, adolescent health policies aim to reduce new HIV infections by 40%, and HIV-related deaths by 20%. They suggest increasing availability of adolescent-friendly provider-initiated testing and counselling (PITC) and having dedicated services for adolescents to improve retention in treatment and care [5, 8, 9]. Testing for HIV and engaging individuals in care is particularly challenging for vulnerable populations such as street youth, as they are often hard to reach, require time to establish trust, and are generally highly mobile and relatively marginalized. [10].

Street-connected children and youth (SCY) spend the majority of their time living and working on the streets. For these individuals, the street is a central reference point, playing a significant role in their daily lives [11]. Data on HIV seroprevalence among SCY in low- and middle-income countries are sparse, but studies in Kenya suggest that high HIV prevalence among SCY [12, 13] exceeds that of other adolescents in the country. In Eldoret, Kenya, we found a HIV prevalence of 6% overall and 15% among females in a study of 120 SCY aged 12–21 [13]. In Kisumu, Kenya, HIV prevalence among street boys was reported to be 4.1% [12], but this study included no females. In contrast, HIV prevalence among Kenyan adolescents aged 15–25 is 2.1% [5]. Additionally, a study on the causes of death of SCY in Eldoret found that HIV was the underlying cause in 37% of 100 documented deaths, including 59% of the 34 females [14]. SCY are at high risk of substance use, sexually transmitted infections, unwanted pregnancies, and physical, sexual, psychological, and economic exploitation and violence [13, 15–21]. They are generally sexually active and likely to engage in high-risk sexual activity, increasing their risks of acquiring HIV [18, 20, 22–27]. Therefore the most common modes of transmission among SCY are probably heterosexual intercourse and sexual violence [12, 28–30]. In addition to the high-risk environment and burden of HIV, SCY experience barriers in accessing care and services due to their marginalization, stigmatization by community and healthcare providers, and high levels of mobility [31, 32].

Interventions involving peers have been used to improve engagement in care in hard-to-reach populations and may overcome many of the barriers faced by the SCY community [33]. A systematic review conducted by Genberg *et al.* found some effect of peer interventions in improving linkage and retention, but reported mixed findings on ART adherence, viral suppression, and mortality. Peers who are employed to improve outcomes related to HIV are generally seropositive and receiving treatment themselves. Due to their lived experiences, peers can create a shared social identity and engage individuals in HIV prevention, care and treatment interventions through education, offering support, and providing referrals as needed [34]. Peer navigators (PNs) can also assist HIV-positive individuals in overcoming logistical difficulties in accessing services as well as maintaining engagement in care [35]. Given the burden of HIV discovered in SCY in Eldoret, Kenya, we sought to pilot and evaluate the use of PNs to promote uptake of HIV testing, initiation of treatment and retention in care among an observational cohort of SCY in Eldoret, Kenya.

## Methods

### Study Setting

Uasin Gishu County (UG) is one of 47 counties in Kenya. According to the most recent census, the total population of UG stood at 894,179, of whom 41.5% were aged 14 years or under [36]. Eldoret city, the administrative capital of UG, has a population of 289,380 [37]. Located approximately 350 km north-west of Nairobi, it is home to Moi University, Moi Teaching and Referral Hospital (MTRH), and the Academic Model Providing Access to Healthcare (AMPATH), a large PEPFAR-funded HIV care and treatment program described elsewhere and headquartered at MTRH in Eldoret [38, 39].

### The Peer Navigator Program

The PN program was launched as a care initiative in response to the HIV epidemic identified among SCY in Eldoret. Two PN positions, one male and one female, were advertised through AMPATH, and the successful candidates were employed from January 2015. A PN was initially defined as a person living with HIV and aged between 18–24 years, who had greater than 1 year of recent experience being street-connected. Sometime after the program started, it was discovered that one was actually HIV-negative. We quickly realized the advantage of having PNs of mixed serostatus (one positive, one negative) in an attempt to increase inclusivity and reduce HIV-related stigma of the PNs or PN program. The PNs were required to be individuals known by the street community, who knew where and how to reach SCY, and had literacy and numeracy skills to Standard/Grade 4 level. The PN position required individuals to be able to conduct community outreach and follow-up individuals over time. The job description explicitly stated that the purpose of the position was to increase linkage to HIV testing and treatment for the SCY population, and to offer education and support on an ad hoc basis to SCY in Eldoret about HIV and related issues. The program was launched in January 2015 after sensitization and mobilization through community meetings known locally as *‘mabaraza’* [40], traditional community assemblies, group and individual discussions with SCY and other opinion leaders in the community. The initial PN training took place over 5 days and was comprised of extensive multi-disciplinary training by AMPATH clinicians for the PNs on HIV/AIDS prevention, treatment and care, reproductive health, counselling, documentation, as well as navigation through the AMPATH HIV linkage and treatment process. This was followed by intensive mentoring and supervision by the social worker. On-going supervision, on-the-job training, and training courses by AMPATH clinicians on reproductive health and ART were provided for the duration of the program. The PNs were based at the adolescent HIV and reproductive health clinic at MTRH.

### Field Engagement and Procedures

SCY primarily reside in specific locations on the street (known locally as ‘barracks’). The PNs undertook extensive outreach and sensitization at these locations to establish rapport and build trust with SCY. This was aided by a pre-existing relationship between the research team and the SCY community in Eldoret [41, 42]. The Youth Charter in Kenya defines adolescents as individuals aged between 15 and 30 years of age [43, 44]. For this program, the PNs engaged male and female SCY who were defined as individuals aged < 30 years who either (a) spent the majority of both days and nights on the street and had limited-to-no parental/guardian contact or (b) spent a portion or majority of their time on the street and had a parent/guardian/caregiver who they returned to at night. Individuals older than 29 were able to receive services but were not included in the analysis. SCY were recruited to the program regardless of their HIV status or current enrolment in care. The female PN focused outreach on locations where female SCY were known to frequent to ensure representation of this especially hard to reach population, as the female SCY were more comfortable interacting with a female PN. As time went on, SCY of all ages became comfortable interacting with both the male and female PNs. SCY interested in engaging with the PN, whether HIV status was known or not, either arrived at the clinic by themselves, or were accompanied by the PNs from town for their initial encounter or at a subsequent follow-up encounter. An encounter was defined as a meeting with the PN where data were collected using the PN encounter form. Engagement into the program was on a continuous basis, with PNs vistiing the barracks multiple times a week throughout the program duration. SCY who initially declined to participate were free to engage with the PN program when they chose.

At an initial encounter, the PN completed an initial encounter data collection form, discussed HIV prevention and offered condoms, assessed HIV status, and, if appropriate, offered HIV counselling and linkage to HIV testing services at AMPATH by Ministry of Health certified HIV counsellors. Those who tested negative were encouraged to return for a general follow-up encounter every 6 months. During each follow-up they were asked whether they had tested for HIV elsewhere since the last encounter. SCY who disclosed their HIV-positive status at their initial or follow-up encounter were not re-tested unless they requested it. Individuals who tested HIV-positive were offered monthly HIV care encounters with the PN, were asked about engagement in HIV care and were linked (or re-linked) to care at AMPATH, where they were encouraged to initiate ART and attend regular appointments with the AMPATH healthcare providers. During these monthly care encounters the PNs offered to accompany the SCY from town to the AMPATH clinicians. They also completed a brief encounter form collecting any change in sociodemographic information as well as engagement in HIV services and reasons for not taking ART if applicable. The PNs supported linkage and engagement in HIV services by making SCY feel comfortable as they were in the presence of peers, assisting them with scheduling and reminding them about clinic appointments, providing emotional support and information, as well as navigation and accompaniment through the process of the appointment, e.g. seeing different clinicians in different buildings. Follow-up of those who were HIV-positive was monitored using a diary and automated reminders from the AMPATH Medical Records System (AMRS). For those who were not diagnosed with HIV, follow-up was monitored by the PNs asking individuals when they had last been tested during their visits to the barracks. The PNs referred SCY to HIV-related and other services as needed. HIV testing, ART and follow-up appointments were provided free of charge as part of the AMPATH HIV program. A waiver was obtained from MTRH for all fees and charges related to care and treatment for many non-HIV services including hospitalization if necessary.

### Sources of Data

This study utilized routinely collected clinical care data from the PN program as well as the AMRS. At each encounter, the PNs interviewed SCY privately and completed structured initial, general follow-up, or care encounter forms. Sociodemographic information, living situation, female reproductive health, general health issues, HIV testing and uptake of care, and referrals were collected at each time point.

Sociodemographic variables included age, gender, location, ethnic group, living situation including sleeping location (in a shared shelter, on the street on a veranda or by a kiosk, in a house, or at the barracks/base) and who they stay with at night (with parents, other family, spouse, boyfriend, girlfriend, alone, or other). Data on female reproductive health included current and previous pregnancy status, if pregnant antenatal care attendance, and whether they stay with their own or someone else’s children. SCY were also asked about common symptoms including those of sexually transmitted infections (STIs), cough, fever, and tuberculosis diagnosis, symptoms, and treatment. Information collected on HIV testing included whether they had ever been tested for HIV, if they knew their status, were willing to get an HIV test during that encounter, and result of the test. Those who knew their HIV-positive status or who newly tested HIV-positive were asked whether they had ever been to, were currently in, or were willing to go for HIV care. Information on willingness to work with a PN, ART use, and disclosure of HIV status were also collected for individuals who were HIV positive.

### Data Analysis

Primary outcomes for this evaluation were uptake of HIV testing, initiation of ART, and retention in care. Retention in care was defined as having attended a clinic visit within 90 days of the expected follow-up date. Individuals who did not attend within the 90 days were defined as lost-to-follow-up (LTFU). Data collected were entered into EpiInfo (version 6), and exported into STATA.12 and R for analysis. Individuals over 29 years of age were excluded from analysis. Categorical variables were summarized using frequencies and corresponding percentages, and compared using the Chi square test or Fisher’s exact test. Continuous variables were summarized using mean/standard deviation or median/interquartile range, and compared using the Student’s t-test or Mann-Whitney U test. The analysis was stratified by sex, and infants perinatally exposed to HIV were excluded from the analysis of HIV outcome variables. Trends prior to and after ART initiation were compared graphically. Time to ART initiation and time to LTFU was studied using Kaplan Meier survival function and compared by care enrolment category (pre-PN or PN). Follow-up time was defined as days from enrolment with PN to the last AMPATH HIV care clinic visit date prior to the closure of the database for analysis.

## Results

From January 2015 to October 2017, the PNs engaged a total of 817 individuals from the street community in Eldoret. Of these, 781 were aged 29 and below, and 36 were aged 30 and above (these individuals were excluded from further analyses). The sociodemographic characteristics of participants are shown in Table 1. The median age of those included in the analyses was 16 (IQR 13–20), with 51.3% (n = 401) aged between 15–24 years. A quarter (25.1%) were female. On average, males were younger than females (median 15, IQR 13–19 vs. 18.5, IQR 14–23, p < 0.001). Most males lived on the street or in rented houses with friends, whereas females predominantly spent their nights in a rented house either with a spouse/boyfriend, friends, or other family members. Only 8.5% of SCY reported living with parents, with females being almost three times more likely to do so than males.

**Table 1:** Background characteristics and living situation of eligible SCY

Variable	Total N = 781	Male N = 585 (74.9)	Female N = 196 (25.1)	P value
Age				
< 5	16 (2.1)	4 (0.7)	12 (6.1)	< 0.001
5–9	26 (3.3)	17 (2.9)	9 (4.6)	0.255
10–14	249 (31.9)	217 (37.1)	32 (16.3)	< 0.001
15–19	256 (32.8)	204 (34.9)	52 (26.5)	0.031
20–24	145 (18.6)	92 (15.7)	53 (27.0)	< 0.001
25–29	79 (10.1)	47 (8.0)	32 (16.3)	0.001
Missing	10 (1.3)	4 (0.7)	6 (3.1)	0.010
Living situation				
On the street	411 (52.6)	375 (64.1)	36 (18.4)	< 0.001
In a shelter/house	308 (39.4)	170 (29.1)	138 (70.4)	< 0.001
Barracks/base	35 (4.5)	29 (5.0)	6 (3.1)	0.267
Missing	27 (3.5)	11 (1.9)	16 (8.2)	< 0.001
Living with^a^				
Parents	66 (8.5)	32 (5.5)	34 (17.4)	< 0.001
Other family^b^	40 (5.1)	16 (2.7)	24 (12.2)	< 0.001
Friends	474 (60.7)	429 (73.3)	45 (23.0)	< 0.001
Spouse, boyfriend, or girlfriend	88 (11.3)	31 (5.3)	57 (29.1)	< 0.001
Alone	71 (9.1)	59 (10.1)	12 (6.1)	0.095
Other^c^	8 (1.0)	4 (0.7)	4 (2.0)	0.102
Missing	38 (4.9)	16 (2.7)	22 (11.2)	< 0.001

^a^Sum of categories is greater than 781 because some SCY live with people/groups from more than 1 category

^b^Other family included siblings, grandparents, aunts, and uncles

^c^Other living partners included single mothers and children

### HIV Testing and Prevalence

Table 2 presents the characteristics of HIV testing in the SCY engaged in the PN program. Just under half (47.8%) of the SCY stated that they had tested for HIV before their first contact with the PNs. Of these, 21 males and 31 females (3.6%, vs. 15.8% p < 0.001) reported their HIV-positive status at the initial encounter. Consent to and uptake of HIV counselling and testing among the others at the initial encounter was very high (88.8%, n = 647), though females were less likely than males to consent to testing (80.0% vs. 91.3%, p < 0.001). Through testingat the initial encounter, 6 male and 6 female SCY (1.1% vs. 3.6%, p < 0.05) were newly diagnosed HIV-positive.

**Table 2:** HIV testing

Variable		Total N = 781	Male N = 585 (74.9)	Female N = 196 (25.1)	P value
Ever been tested for HIV	Yes	373 (47.8)	244 (41.7)	129 (65.8)	< 0.001
	No	303 (38.8)	263 (45.0)	40 (20.4)	< 0.001
	Refuse to Answer	92 (11.8)	75 (12.8)	17 (8.7)	0.119
	Missing	13 (1.7)	3 (0.5)	10 (5.1)	< 0.001
Status at initial encounter (self-stated)	Positive	52 (6.7)	21 (3.6)	31 (15.8)	< 0.001
	Negative	313 (40.1)	217 (37.1)	96 (49.0)	< 0.05
	Don’t know	406 (52.0)	344 (58.8)	62 (31.6)	< 0.001
	Missing	10 (1.3)	3 (0.5)	7 (3.6)	0.001
Tested for HIV with PNs if HIV status unknown or negative					
At initial PN encounter	# Tested^a^	647 (88.8)	515 (91.3)	132 (80.0)	< 0.001
	# Newly HIV positive^a^	12 (1.7)	6 (1.1)	6 (3.6)	< 0.05
At follow up encounter	# Tested^b^	128 (84.2)	94 (83.2)	34 (87.2)	0.555
	# Newly HIV positive^b^	3 (2.0)	0	3 (7.7)	< 0.005
Infants, seroconversion status uncertain or negative - excluded from total HIV positive		4 (0.5)	0	4 (2.0)	0.001
Total HIV positive	All ages	63 (8.1)	27 (4.6)	36 (18.4)	< 0.001
	Age 1–9	7 (0.9)	4 (0.7)	3 (1.5)	0.276
	Age 10–14	9 (1.2)	6 (1.0)	3 (1.5)	0.566
	Age 15–19	10 (1.3)	4 (0.7)	6 (3.1)	0.010
	Age 20–24	16 (2.1)	6 (1.0)	10 (5.1)	0.000
	Age 25–29	21 (2.7)	7 (1.2)	14 (7.1)	0.000
Linked to care of those HIV positive^c^		58 (92.1)	25 (92.6)	33 (91.7)	0.893

^a^N = 729, the total number of people whose stated HIV status was negative or unknown. Individuals who disclosed HIV positive status at initial encounter are not included in counts for HIV testing

^b^N = 152, the total number of people who came for follow-up visits; 113 for males 39 for females

^c^N = 63, the total number of HIV positive SCY

Of the 781 SCY seen by PNs for an initial encounter, 152 (19.5%) individuals who were HIV-negative at the initial PN encounter returned for at least one follow-up HIV test. The majority of returnees had either had an HIV test since their first visit or agreed to have one at the follow up visit. Three females who were seronegative at the initial encounter were found to be HIV-positive at follow-up. Of these, two tested positive through the follow-up PN encounter itself, and one had tested positive elsewhere. No males were found to be HIV-positive at follow-up. Four infants were recorded as being HIV-exposed at the initial PN visit. Two were subsequently found to have not seroconverted, and two had an uncertain HIV status. All four were excluded from further analysis.

Overall, 63 SCY (27 males and 36 females) were HIV-positive, bringing the prevalence of HIV in our sample to 8.1%, with females being four times more likely to be HIV-positive than males (18.4% vs. 4.6%, p < 0.001). At their initial and monthly encounters, HIV-positive SCY were asked ‘whether you would like a PN to remind you to come to your next AMPATH appointment’. Individuals answered ‘yes’ to this question in 42 out of 60 (70%) of initial and 187 out of 203 (92.1%) monthly care encounters. Of the 36 HIV-positive females, two were pregnant at the initial PN encounter, and 20 had ever been pregnant at least once. We were unable to assess whether vertical transmission of HIV took place in these cases.

### HIV Treatment: Linkage to Care, Uptake of ART, Retention in Care, and Viral Load Suppression

**Fig. 1:**
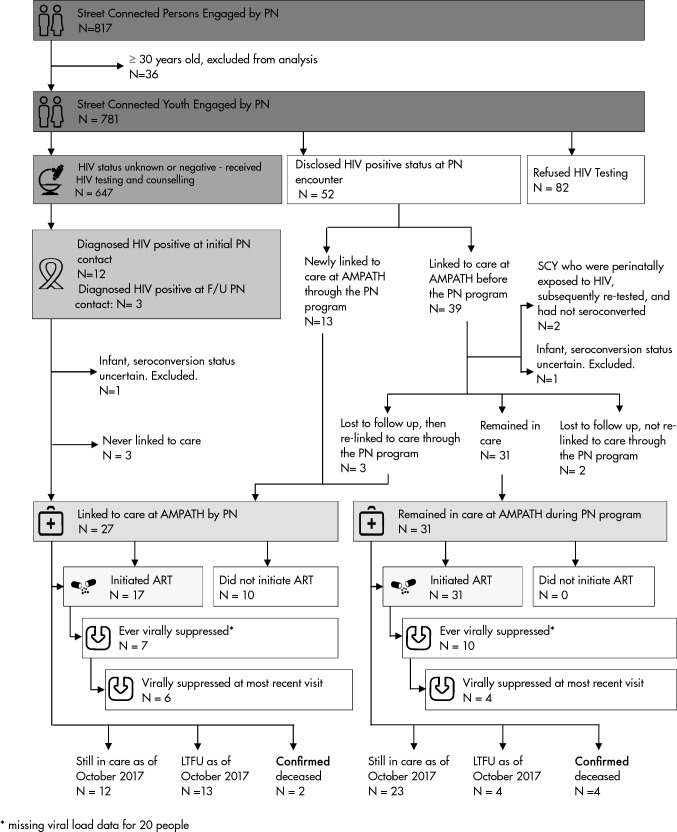
The HIV care cascade for SCY engaging with PNs

**Fig. 2:**
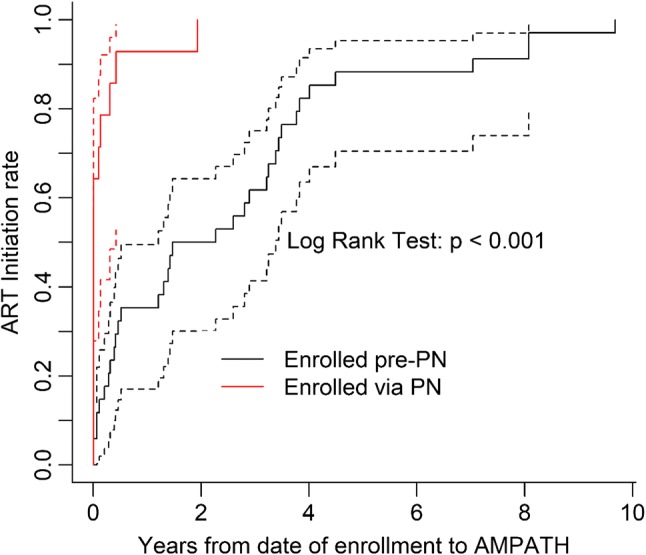
Kaplan-Meier survival curve of time to ART start stratified by type of enrollment

**Fig. 3:**
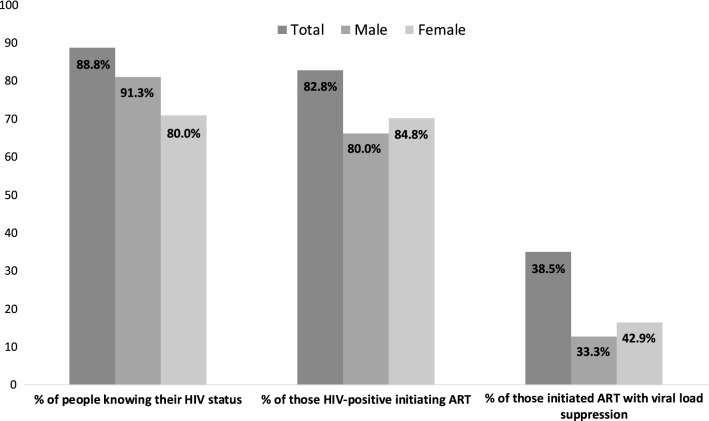
Progress of SCY in PN program toward achieving UNAIDS 90-90-90 target

Figure 1 summarizes the flow of the 781 SCY engaged in the PN program through the HIV care continuum. Of the 63 SCY who were HIV-positive, 25 (92.6%) male SCY and 33 (91.7%) female SCY, were linked—that is, had at least one clinical encounter with an HIV care provider at AMPATH. While the PNs were directly involved in linking 27/63 (43%) HIV positive SCY to care, either for the first time or after previous loss to follow-up (LTFU), 31/63 (49%) SCY encountered by the PN had already linked to care and 27 had initiated ART at some point. The SCY already linked to care were offered the same services as the SCY newly linked to care, specifically support in scheduling and attending follow-up visits. Figure 2 illustrates that SCY engaged by the PNs had a shorter time to initiation of ART after enrolling in care than those enrolled prior to the introduction of the PN program (p < 0.001). Table 3 presents the care outcomes of the 58 SCY who were HIV-positive and linked to care. As of October 2017, 48 (82.8%) initiated ART, 35 (60.3%) were still in care, 17 (29.3%) were LTFU, and 6 (10.3%) were deceased. Of those that were LTFU (n=17), the median time to LTFU was 286.5 days (IQR 198.3–608.5). Of those who did not know their status prior to PN engagement, 15 were diagnosed HIV-positive. Of these, 12 were linked to care, of whom 9 (75%) initiated ART, and 6 (50%) were retained in care. Figure 1 and Table 3 describe ART initiation among SCY who were linked to care, and Fig. 3 shows the progress of SCY in the PN program toward achieving the UNAIDS 90-90-90 target [3].

**Table 3:** HIV treatment and care

Variable	Total N = 58	Linked to care prior to PN engagement				New linked to care by PN			
		Total N = 31	Male N = 13 (41.9)	Female N = 18 (58.1)	P value	Total N = 27	Male N = 12 (44.4)	Female N = 15 (55.6)	P value
Enrolled in AMPATH prior to PN program, and remained linked to care during PN program	34 (58.6)								
On ART prior to PN program^a^	27 (79.4)								
Lost to follow up before PN program but reconnected via PN^a^	3 (8.8)								
Ever started ART	48 (82.8)	31 (100.0)	13 (100.0)	18 (100.0)		17 (63.0)	7 (58.3)	10 (66.7)	0.706
Time to ART (years)	0.5 (0.1, 3.2)	2.3 (0.4, 3.6)	1.4 (0.1, 3.3)	3.1 (0.7, 3.7)	0.109	0.008 (0.003, 0.312)	0.101 (0.003, 0.809)	0.005 (0.003, 0.265)	0.680
Still in care as of end of October 2017	35 (60.3)	23 (74.2)	10 (76.9)	13 (72.2)	> 0.999	12 (44.4)	6 (50.0)	6 (40.0)	0.707
Time to loss to follow-up (days)	286.5 (198.3, 608.5)	286.5 (198.3.0, 608.5)	302.0 (213.0, 604.0)	271.0 (182.0, 610.0)	0.739	251.0 (198.3, 482.8)	261.3 (211.3, 311.0)	251.0 (191.8, 623.5)	> 0.999
Virally suppressed at the last visit (among those alive and on ART^b^	10 (38.5)	4 (20.0)	2 (20.0)	2 (20.0)	> 0.999	6 (100)	2 (100)	4 (100)	N/A
Missing viral load data at last visit^c^	20 (41.7)	7 (25.9)	0 (0.0)	7 (41.2)	0.026	4 (40.0)	3 (60.0)	1 (20.0)	0.229

^a^N = 34, the total number of people who enrolled in AMPATH prior to the PN program and were still in care at some point during the PN program

^b^N = 26, the total number of people with viral load data who are still alive and in care (out of the total 48 who initiated ART, 20 are missing viral load data and 2 individuals with viral load data were LTFU at close of dataset)

^c^N = 48, the total number of people who initiated ART

As shown in Fig. 1, of the 48 HIV-positive SCY who had initiated ART, viral load measurements were available for 28 (58.3%) individuals. Of these, 17 (60.7%) had been virally suppressed at some point during their treatment. The median time to ever achieving viral suppression was 1.7 years (IQR 0.8–2.0). There was no significant difference in time to ART initiation, viral suppression, or lost to follow-up between males and females. As of October 2017, 35 of the 58 individuals living with HIV who had been linked to care were retained in care. However, out of the 26 individuals who had a viral load result and were still alive and in care, only 10 were virally suppressed.

## Discussion

The key findings of this pilot to engage street connected youth in Eldoret, Kenya in HIV testing, treatment and care were as follows. Firstly, high numbers of SCY engaged with the PN and accepted HIV counselling and testing. Secondly, there was a high prevalence of HIV among SCY engaging with PNs, especially among female SCY. This is significantly higher than the background adult HIV prevalence in the region [9]. Finally, high numbers of HIV-positive SCY initiated treatment, linked to care, and were still in care as at the end of April 2017.

A number of factors likely contributed to the PN’s success in engaging SCY in testing and linking them to care. The PNs were known and trusted by SCY, and reached out to SCY at the locations where they congregate and live. A large majority of the SCY engaged by the PNs accepted HIV testing, higher than national averages in adolescent populations in SSA [45]. This was potentially a result of the seamless transition from encounter to testing—immediately after the PN encounter, SCY who accepted HIV testing were escorted by a PN to the clinic. Whilst uptake of HIV testing was high among both genders, female SCY were less likely than male SCY to accept HIV testing. This is a serious concern, since HIV prevalence among females living on the street is higher than that among males. It is also worth noting that the majority of female HIV-positive SCY reported a current or previous pregnancy, which suggests that comprehensive and accessible reproductive care for SCY is critical to preventing vertical transmission of HIV.

The majority of SCY in this sample initiated ART, and time to ART initiation was significantly shorter for SCY who linked to care under the PN, although it is unclear whether this was caused by the PN program or due to a change in policy whereby all patients testing HIV-positive initiated ART regardless of CD4 count. Only a third of participants ever achieved viral load suppression despite relatively high rates of linkage to care or ART initiation. It was not possible to collect data on ART adherence directly, but this finding suggests that adherence to ART is a challenge in this population. The low rate of suppression may also be related to acquired or developed drug resistant viral mutations, causing a reduced response to ART. Systematic reviews measuring ART adherence among general adolescent populations in Africa have reported 81–84% adherence [46, 47]. These rates are higher than those suggested here, reflecting the unique social and economic barriers to care faced by SCY. Youth with multiple barriers to care, including unmet mental health needs, homelessness, and poverty, have been shown to be less able to adhere to treatment [32]. They also may respond to these barriers in ways that negatively affect treatment outcomes—for example, not taking medication in order to conceal their HIV status from others. All these factors may be related to the low levels of viral suppression among SCY in Eldoret [32].

Although approximately half of our cohort were retained in care at the end of the reporting period, just under one third were LTFU. This may be related to instability in the population of SCY in Eldoret, or a variety of social and systemic barriers to retaining these young people in care. Prior research has shown that this population experiences a wide range of challenges, including poverty, high levels of transience, and stigma related to being street-involved [19, 20, 39, 48, 49]. SCY may not have regular meals or a place to store their ART, and are in danger of stigma and discrimination from the street community if found to be HIV-positive [20, 21, 50]. Once SCY leave the hospital grounds, it is difficult to contact them about follow-up visits as the vast majority do not own a mobile phone. Out of fear of police violence [15], many may also change their whereabouts frequently and thus could be difficult to locate. In order to increase adherence and retention, future research could work on identifying structural supports such as meals, access to community-based and/or population-specific care, transport, and ART adherence supports.

Considering the high prevalence of HIV and the multifaceted barriers to HIV testing and treatment facing SCY, PNs may play a crucial role in containing the HIV epidemic among SCY in high HIV burden, low income settings. To the best of our knowledge, this is the first study to report outcomes of a peer engagement project with SCY in SSA to promote uptake of HIV testing, treatment, and care. Our results indicate that even though PNs were highly effective in linking SCY to testing and ART initiation, new strategies are needed to improve viral suppression. These results add to the systematic review carried out by Genberg et al., which found that peer interventions improved linkage and retention, but reported mixed effects on ART adherence and viral suppression [33]. Our cohort may have nearly achieved the first target of 90% of those living with HIV knowing their status, and is close to meeting the second target of the UNAIDS 90-90-90 framework, but more work is needed to achieve viral suppression in 90% of the population [3].

A strength of this study is that it was rooted in AMPATH, a large and well-established HIV program with strong links to HIV testing and care centres in a medium-sized city in a low-income setting. Secondly, this study focused on a particularly vulnerable and hard to reach population of adolescents and youth. This pilot contributes to the development and evaluation of evidence-based programs and interventions to address the burden of HIV in this high-risk community, especially among adolescent girls and young women. Finally, during the period of data collection for this study, a number of related initiatives took place which may have promoted contact between SCY and PNs or healthcare professionals, including the opening of the MTRH-Rafiki Center for Excellence in Adolescent Health (November 2016), a Point in Time count of SCY in Eldoret (September 2016) [51,] and a voluntary male medical circumcision and educational ‘coming-of-age’ retreat for SCY (December 2016–May 2017) [52, 53.] These initiatives extended the program’s reach in the SCY community and in connecting PNs to HIV-positive SCY looking to enter care. Limitations of this study include its reliance upon self-reported data recorded chiefly by the PN. We cannot rule out some selection bias if some SCY did not want to approach the PNs and thus were never included. Data quality also may have been limited by some inconsistencies in collection of data and low literacy levels in the target population. However, data quality did improve over time as the PNs built trust among SCY and became more experienced in data collection. As this study did not have a control group, it was only possible to measure uptake of the intervention rather than effectiveness. Furthermore, missing viral load data limited our ability to assess the impact of the PN program on viral suppression.

## Conclusion

This pilot study suggests that PNs in this context appear to be feasible and potentially effective at promoting uptake of HIV counselling, testing, care and treatment. However, more research is needed to evaluate the optimal role of PNs in linkage and retention, and improve ART adherence in this population. Innovative, holistic programs may be necessary to address multifaceted barriers to long-term HIV treatment in the most vulnerable populations, potentially including targeted programs for SCY.
